# In Vitro and In Silico Anti-Rheumatic Arthritis Activity of *Nyctanthes arbor-tristis*

**DOI:** 10.3390/molecules28166125

**Published:** 2023-08-18

**Authors:** Ayushi Sharma, Anjana Goel, Zhijian Lin

**Affiliations:** 1 Department of Biotechnology, GLA University, Mathura 281406, India; ayushi.sharmabsc15@gmail.com; 2 Department of Clinical Chinese Pharmacy, School of Chinese Materia Medica, Beijing University of Chinese Medicine, Beijing 102488, China

**Keywords:** rheumatoid arthritis, Harshringar, anti-oxidant, anti-arthritic, ADMET

## Abstract

Rheumatoid arthritis (RA) is an autoimmune disease characterized by bone and joint degeneration. Existing anti-inflammatory chemotherapy drugs offer temporary relief but come with undesirable side effects. Herbal medications have shown positive effects on RA symptoms with minimal adverse reactions. In this study, we investigated the potential of *Nyctanthes arbor-tristis* (NAT) through in vitro and in silico research. Hydroethanolic extracts of harsingar were prepared using the reflux method, containing alkaloids, phenol, saponin, steroids, proteins, tannins, terpenoids, carbohydrates, glycosides, and flavonoids, which exhibited TPC (98.56 ± 0.46 mg GAE/g) and TFC (34.51 ± 0.45 mg CE/g). LC–MS/MS analyzes the active compounds in the extract. NAT exhibited the best scavenging capabilities at 1 mg/mL in anti-oxidant and anti-arthritic activity. Maximum splenocyte proliferation occurred at 250 µg/mL. In vitro cell splenocyte studies revealed the downregulation of TNF-α and the upregulation of IL-10. Additionally, an in silico study demonstrated that bioactive constituents and targets bind with favorable binding affinity. These findings demonstrate the potential of Nyctanthes arbor-tristis in exerting anti-arthritic effects, as supported by in vitro and in silico studies. Further mechanistic research is necessary to validate the therapeutic potential of all phytoconstituents in RA treatment.

## 1. Introduction

Rheumatoid arthritis (RA) is an autoimmune, inflammatory, chronic disease linked with inflammation of the synovial and joint membranes. It also causes cartilage destruction, discomfort, and deformation in the bone cartilage. Inflammatory mediators, in particular, have been known to play a critical role in inflammation, stiffness, and impairment during the onset of RA. It is an autoimmune disease of the synovial joints that is constantly brought on by inflammatory mediators and infections [1]. Every year, 0.5% of the world’s population is affected by rheumatoid arthritis, with women outnumbering men by a factor of three [2]. RA is strongly linked to an auto-immune response triggered by a variety of environmental factors (such as microbiome diversity, the virus rubella, etc.), epigenetics, and genetics. There are three phases of RA progression: inflamed synovium; discomfort and swelling; and finally, cartilage and bone degradation that cause joint injury [3]. TNF-α (tumor necrosis factor), interleukin-1b, enzymes of lysyl oxidase (LOX), cyclooxygenase II (COX-II), reactive oxygen species (ROS) of NO, prostaglandin-endoperoxide synthase (PTGS), prostaglandins, H_2_O_2_, TGF (transforming growth factor), and MCSF (macrophage colony-stimulating factor) are only a few of the many proinflammatory cytokines that have a role in RA [4].

Allopathic medicines have gotten better over the years; they have been shown to help slow the progression of diseases by relieving symptoms and improving the quality of life of those who have been hurt by them [5]. However, using synthetic medications for an extended period of time has resulted in numerous negative side effects for the patient [6]. Certolizumab pegol is an FDA-approved drug that has been used as an immunosuppressant in RA [7]. The drug, on the other hand, has toxic effects, increasing the risk of urinary tract infections and respiratory infections [8]. Similar to how extended use of RA medications may lead to complications like pneumonia and TB, long-term use of other medications has also been linked to serious side effects in human embryos [9].

Herbal medicines are emerging as safe alternatives to such harmful diseases. They do not show as many adverse effects on the human body as synthetic drugs. Over 2500 plant species are currently used as herbal medicines in India [10]. Furthermore, many crude herbal-based drugs and their constituents have high anti-oxidant activity and scavenge free radicals, both of which promote cartilage damage and inflammatory responses [11]. Natural chemical constituents extracted from medicinal plants can interact with and modulate the expression of pro-inflammatory signaling on the inflammation pathway, thereby reducing the arthritic effect [12].

*Nyctanthes arbor-tristis* Linn is a member of the Oleaceae family and is commonly known as night jasmine. It is a species of Nyctanthes native to Southeast Asia and South Asia. In India, the plant is found to be grown in the Himalayas and has been found in tracts of Nepal to the east of Assam, Tripura, Bengal, Jammu, and Kashmir, extending up to Godavari’s central region in the south [13]. The plant has high medicinal potential in Ayurveda. Nyctanthes have been identified as a reservoir of beneficial chemical entities that could be used as medicines, intermediates to investigate newer molecules, and the most recent leads for drug synthesis in modern times [14]. Previously, therapeutical analysis showed that this plant possessed antimicrobial, anti-oxidant, antiviral, antidiabetic, antimalarial, antifungal, anti-inflammatory, anticancer, CNS depressant, hepatoprotective, and immunostimulant activities [15]. All the parts of the plant have been found to have several potential pharmacological entities, and this has been scientifically recognized by in vitro and in vivo studies. The decoction and juice of plant leaves have been used in conventional drug systems to treat inflammatory disorders, arthritis, and rheumatism [16]. The flowers of this plant are useful in treating piles and several skin diseases because they are antibilious, carminative, stomachic, and astringents for the bowel. Conventionally, powdered stem bark was used by the RA to obtain relief from rheumatic joint pain [17]. Even though the plant has been extensively used as a remedy for rheumatic pain, the scientific knowledge regarding its usage is very limited [18]. Given the medicinal potential of *Nyctanthes arbor-tristis*, an extensive study has been conducted to investigate the plant’s anti-inflammatory and analgesic properties. The current study was conducted to demonstrate the medicinal potential of hydroethanolic extract of NAT and to discover its compelling anti-inflammatory effects by in vitro and in silico approaches. 

## 2. Results

### 2.1. Phytochemical Screening

#### 2.1.1. Qualitative Phytochemical Screening

Qualitative phytochemical screening of *Nyctanthes arbor-tristis* extract showed the presence of alkaloid, phenol, saponin, steroids proteins, tannins, terpenoids, carbohydrate, glycosides and flavonoids.

#### 2.1.2. Total Flavonoids Content and Total Phenol Content

The NAT extract was assessed to determine total phenolic content (TPC) and total flavonoid content (TFC). The results indicated that the NAT extract exhibited TPC (98.56 ± 0.46 mg GAE/g) and TFC (34.51 ± 0.45 mg CE/g).

#### 2.1.3. LCMS Analysis

**Figure 1:**
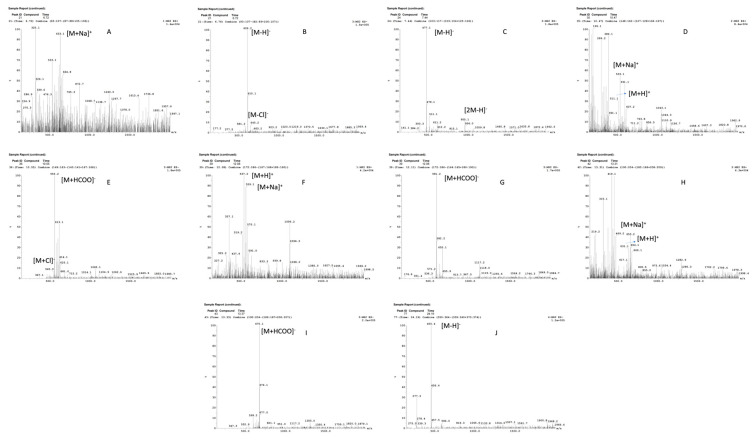
(**A**,**B**): Mass spectrum of compound 1 at Rt 6.7, M.W 610; (**C**): Mass spectrum of compound 2 at Rt 7.4, M.W 478; (**D**,**E**): Mass spectrum of compound 3 at Rt 10.47, M.W 510; (**F**,**G**): Mass spectrum of compound 4 at Rt 12.08, M.W 536; (**H**,**I**): Mass spectrum of compound 5 at Rt 13.31, M.W 630; (**J**): Mass spectrum of compound 6 at Rt 24.1, M.W 456. **Abbreviations:** electrospray positive (**ES+**); electrospray negative (**ES−**); retention time (**RT**); hydrogen (**H**); sodium (**Na**), chlorine (**Cl**); molar mass (**mol mass**).

The mass spectrum of the detected compounds is shown in (Table 1 and Figure 1), along with the LC–MS chromatogram of the hydroethanolic extract of *Nyctanthes arbor-tristis*. It was noted that various peaks were obtained at various retention times. 

### 2.2. In Vitro Anti-oxidant Activities

**Figure 2:**
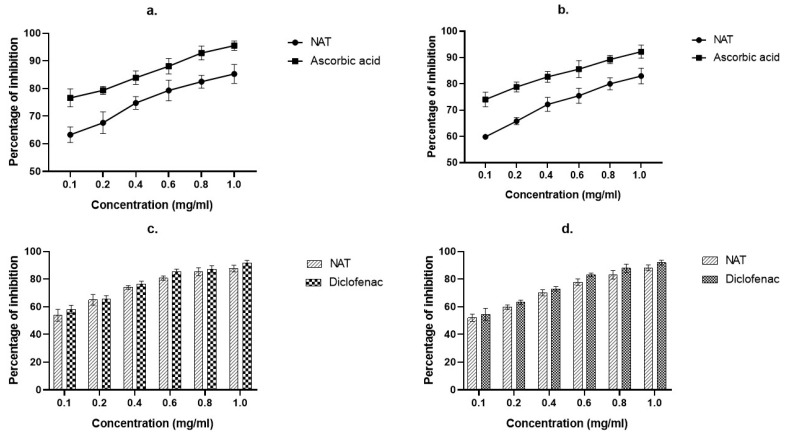
Result of anti-oxidant (DPPH assay (**a**) and H_2_O_2_ assay (**b**) and anti-arthritic (protein denaturation (**c**) and HRBC membrane stabilization (**d**)).

In this study, DPPH and H_2_O_2_ were used to test the crude extract of *Nyctanthes arbor-tristis* for potential anti-oxidant activity, and the inhibitory impact was compared to that of ascorbic acid, the gold standard. The hydroethanolic extract of 1 mg/mL among these concentrations showed significant activity in a dose-dependent manner. In the DPPH assay, the percentage inhibition of 1 mg/mL was determined to be 85.26 ± 3.43%, which was somewhat lower than the activity of ascorbic acid at its standard concentration (95.53 ± 1.71%), and showed higher activity than the other concentrations (Figure 2a). H_2_O_2_ showed 82.98 ± 3.01% inhibitory activity at 1 mg/mL concentration, which was lower than ascorbic acid’s activity (92.24 ± 2.47%). (Figure 2b).

### 2.3. In Vitro Anti-Arthritic Activities

The ability of NAT extract to reduce inflammation was examined using an assay for membrane stabilization and protein denaturation. NAT has demonstrated a favorable outcome in both studies in a dose-dependent manner (Figure 2). In the protein denaturation inhibitory assay utilizing various extract doses, 1 mg/mL showed considerable inhibition with the highest inhibition capacity (87.63 ± 2.43%), which was quite lower than Diclofenac sodium (89.01 ± 1.23%) (Figure 2c). The extract of *Nyctanthes arbor-tristis* showed the greatest stabilizing effect (88.23 ± 2.09%) and was also found to be lower than diclofenac sodium (91.92 ± 1.69%) (Figure 2d).

### 2.4. Effect of NAT Leaves on Splenocytes Proliferation

On the basis of optimization of Con A concentration for splenocyte proliferation, it was discovered that a concentration of 10 µg/mL yielded the best result, and this concentration was subsequently used for further experiments. The in vitro investigation demonstrated that the NAT leaf extract at concentrations of 50, 100, 250, 100, and 1000 µg/mL resulted in 36.97%, 42.74%, 55.66%, 4.97%, and −36.77% proliferation, respectively, in the cultures of spleen cells (refer to Table 2). The study revealed a dose-dependent effect of the NAT extract on the proliferation of splenocytes. Additionally, it was determined that 1000 µg/mL exhibits toxicity and the rest of the concentrations were deemed safe for splenocytes, allowing for their use in subsequent cytokine assays.

### 2.5. Effect of Nyctanthes Arbor-Tristis on Cytokines (TNF-α and IL-10) Induction

Table 3 presents the data regarding the impact of various concentrations of NAT (50, 100, 250, 500, and 1000 µg/well) on TNF-α and IL-10 levels in rat splenocytes following in vitro treatment. Upon examination of the data in Table 3, it was observed that there was a noteworthy increase in TNF-α and IL-10 levels (expressed in pg/mL) in splenocytes compared to the control group across most of the concentrations used. However, it is important to note that this effect did not exhibit a dependency on the concentration of NAT.

### 2.6. In Silico Studies

#### 2.6.1. Preparation of Protein

A PDB file containing the three-dimensional structure of cyclooxygenase 2 (COX-2) protein (PDB ID: 1CVU) and tumor necrosis factor (TNF-α) protein (PDB ID: 6RMJ) was obtained from the Protein Data Bank (RCSB PDB: Homepage). Python Molecular Version was used for the protein preparations (PMV-1.5.7). Bonding arrangements were established after the addition of a heavy hydrogen atom. Even water molecules were eliminated. The structure was finally optimized and reduced in size using a force field. 

#### 2.6.2. Preparation of Ligands 

The ligands’ 2D structures were obtained from the NCBI PubChem database and converted to the PDB format using openbabel. The retrieved ligand structures were minimized for potential interactions with the target proteins COX-2 and TNF for molecular simulations.

#### 2.6.3. Ramachandran Plot

**Figure 3:**
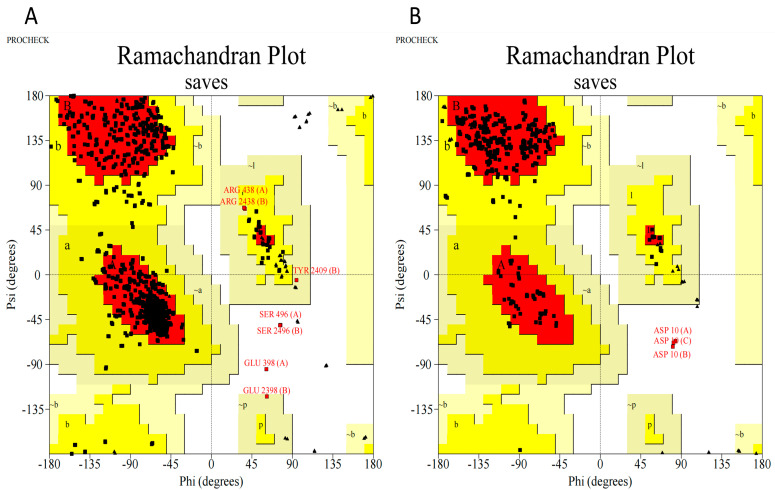
Ramachandran plot (**A**) COX-2 and (**B**) TNF-α. Red color (core), yellow (allowed), and beige (generously allowed). Residues in most favoured regions [A,B,L]; Residues in additional allowed regions [a,b,l,p]; Residues in generously allowed regions [~a,~b,~l,~p].

The Ramachandran plot showed that 87.73% of COX-2 remained in the most favored region, 11.6% remained in the additional allowed region, 0.4% remained in the generously allowed region, and 0.3% remained in the disallowed region; meanwhile, for TNF-α, 90.2% remained in the most favored region, 9.0% remained in the additional allowed region, and 0.8% remained in the disallowed region (Figure 3). As such, the protein is favorable for further study.

#### 2.6.4. Interactions between Bioactive Compounds (Ligands) and Proteins

**Figure 4:**
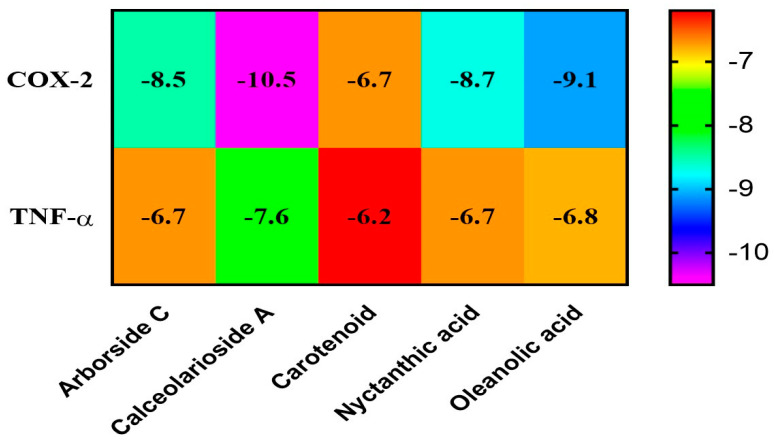
Heat map of interaction between bioactive compounds (ligands) and proteins.

**Figure 5:**
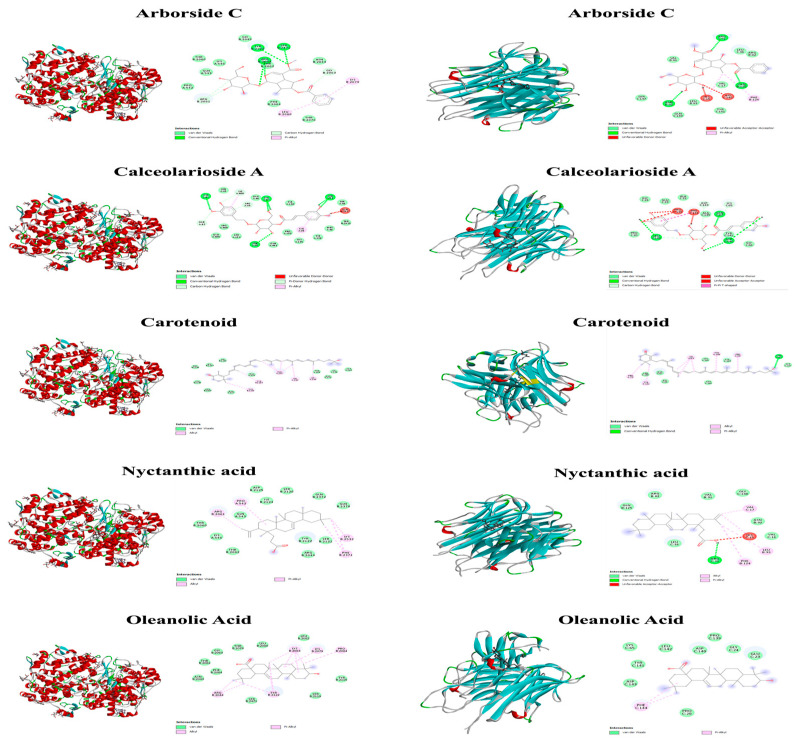
Schematic diagram of TNF-α and COX-2 with phytoconstituents complex interaction using drug discovery studio.

Molecular docking was conducted with Autodock Tools, and the output was the binding energy of the protein to the ligand. Bioactive compounds with the lowest binding energies were considered best in inhibiting the target proteins as shown in Figure 4. The binding energies between acceptors and ligands and the visualization results are listed in Figure 5. A lower value of binding energy shows that the compound has a better and more vigorous affinity with the receptors. The Protein-Ligand Interaction Profiler (PLTP) website was used to analyze how proteins bind to bioactive compounds.

#### 2.6.5. Pharmacokinetic and Bioactivity Properties

The ADME/T test was used to determine the pharmacokinetics and bioactivity of a drug molecule in a living system [19]. The blood–brain barrier is an essential target for drugs that aim to treat neurological disorders [20]. Drugs are typically taken orally, so it is reasonable to assume that the intestinal tract is where they undergo most of their absorption. P-glycoprotein is involved in the intracellular transport of numerous drugs [21]. The drug transport system is thus affected by its inhibition [22].

Drugs taken orally end up in the liver after making their way through the circulatory system. They are metabolized by a set of liver enzymes and then passed out of the body in the urine or in bile. As a pharmacological parameter, drug binding to plasma proteins affects not only the drug’s pharmacodynamics, but also its distribution and elimination. A drug’s effectiveness is determined by how well it binds to proteins in the blood plasma as shown in Table 4.

#### 2.6.6. Prediction of Activity Spectra for Substances (Pass) Prediction Study

Prediction of activity spectra for substances (PASS) study toxicity and LD50 value were not determined by ProTox II because there was not enough information. Phytoconstituents, on the other hand, have an estimated LD50 and toxicity class given in Table 5. The behavior of six planned biological activities with no adverse effects was predicted using the PASS study. The PASS prediction experiment was conducted, and the results with Pa > 0.7 were retained because they are very predictive. Anti-inflammatory, anti-oxidant, immunosuppressant, free radical scavenger, nitric oxide antagonist, and bone disease treatment properties have been observed in conjunction with phytoconstituents. Table 5 displays the outcomes of studies that attempted to predict substances’ activities using the prediction of activity spectra for substances (PASS).

## 3. Discussion

Chronic autoimmune diseases such as RA cause joint redness, swelling, and fatigue, in addition to an increased death rate. To begin with, the synovial fluid lining the joints takes a hit, followed by the cartilage and bone, and finally, it can cause multi-organ failure. Genetic factors, stress, smoking, and viral infections all play a role in increasing arthritis risk, but women are disproportionately affected [23].

Citrullinated proteins, rheumatoid factors, and other self-proteins, like vimentin, a-enolase, histones, fibronectin, fibrinogen, and collagen, are released because of changes that happen after transcription [24]. Anti-citrullinated protein antibodies (ACPAs) are generated because these proteins are not recognized as “self” proteins. Immune complex formation involves multiple intracellular and intercellular signaling mechanisms. Inflammation can now be reduced with the help of extracellularly specific biological DMARDs, like sarilumab and adalimumab. On the other hand, nonsteroidal anti-inflammatory drugs (NSAIDs) merely act as temporary pain relievers, rather than having a long-term mechanistic modification in trying to prevent bone and joint damage [25].

Flavonoids have been shown to have anti-inflammatory, anti-bacterial, anti-allergic, and antiviral activity [26]. Alkaloids with pharmacological effects are used as medications and recreational drugs [27]. Tannins and phenols, which make up the polyphenolic group, are known to have anticancer, anti-oxidant, anti-inflammatory, and antimicrobial properties. Terpenoids in hydroalcoholic extract have anti-arthritic activity, according to [28]. 

LC–MS/MS analysis is one of the first steps in determining the nature of active compounds in medicinal plants. The current study discovered a number of active compounds via LC–MS analyses of a hydroethanolic extract of *Nyctanthes arbor-tristis* leaf, some of which have anti-arthritic/anti-inflammatory activity, which is of interest in these studies (Table 1). 

Denaturation of synovial membrane and tissue proteins is a well-known cause of arthritic diseases. Autoantigens may be produced in certain arthritic diseases due to protein denaturation [29,30]. The possible mechanism underlying denaturation is the alteration of hydrogen, hydrophobic, disulphide, and electrostatic bonds in proteins [30], and during inflammation, lysosome lysis releases toxic enzymes into the circulatory system. NSAIDs are used to prevent the release of lysosomal enzymes by neutralizing lysosomal membrane damage. Unwanted environmental factors, such as hypotonic medium, high temperatures, and chemical medications containing methotrexate, hydroxychloroquine, and diclofenac sodium, may impair RBC membrane integrity and cause hemolysis [31]. Evaluation of the protective function may be helpful in determining anti-inflammatory properties because RBCs mimic the lysosomal membrane. The development of anti-arthritic medications would benefit from agents that prevent protein denaturation and aid in membrane stabilization, and NAT extract is capable of both things, as shown in Figure 2.

Significant (*p* < 0.01) stimulatory effects of NAT on splenocytes were discovered in the current study. It can be concluded that the hydroethanolic extract of NAT may have increased B- and T-cell populations, which are crucial for humoral and cell-mediated responses, based on the findings of earlier studies of hematology [32], immune responses [33], and current splenocyte proliferation experiments. 

The current study shows that NAT has a significantly diminished (*p* < 0.01) regulatory effect on the induction of tumor necrosis factor in in vitro experiments (Table 3). TNF-α is a multipurpose pro-inflammatory cytokine that is mainly secreted by mast cells and macrophages [34]. The use of TNF-α in the treatment of immune disorders has gained attention in recent years. Chronic inflammatory conditions and harmful changes in lipid and glucose metabolism are brought on by TNF-α protracted persistence [35]. The use of biological agents derived from plants to inhibit important inflammatory mediators, like TNF- α, IL-6, and IL-1, requires scientific attention in this crucial era. Infliximab (an anti-TNF antibody), golimumab, etanercept, adalimumab, and certolizumab have all received clinical approval for the treatment of a variety of inflammatory diseases in humans [36]. There is scientific support for using NAT leaves to prevent diseases brought on by chronic inflammation, and NAT treatment of splenocyte cultures was found to significantly (*p* < 0.01) increase IL-10 secretion compared to controls (Table 3). NAT causes a distinct decline in the level of TNF-α in in vitro experiments. By inhibiting the activity of TH1 cells, NK cells, and macrophages, IL-10, an anti-inflammatory cytokine responsible for regulating immune response, prolonged the time it took for pathogens to be cleared [37,38].

Molecular docking of the identified compounds on the active site of COX-2 and TNF -α was studied to find out how they affect the pathway controlled by the COX-2 and TNF -α protein, which is a key mediator of inflammation in diseases like rheumatoid arthritis and osteoarthritis and plays a major role in oxidation and early inflammation, etc. (*p* < 0.01). This study also looked into the anti-arthritic activity of Arborside C (docking score −8.5 and −6.7 kcal/mol), Calceolarioside A (docking score −10.5 and −7.6 kcal/mol), carotenoid (docking score −6.7 and −6.2 kcal/mol), nyctanthic acid (docking score −8.7 and −6.7 kcal/mol), and oleanolic acid (docking score −9.1 and −6.8 kcal/mol), and the effect of these compounds on anti-arthritic activities was also validated in the in vitro studies. According to our findings, the activated components of *Nyctanthes arbor-tristis* have a high potential for new medications in the ADME evaluation. However, the contribution of most of them to anti-arthritic activities should be further uncovered; 60 percent of those who suffer from rheumatoid arthritis look for herbal remedies [39]. According to Chopra et al., when it comes to establishing the legitimacy of traditional herbal medicines on a global scale, more rigorous studies are necessary to determine their efficacy and safety [40]. The main goal of our study was to find new molecules from NAT that showed promise and could be used to treat arthritis with few side effects. A hydroethanolic extract of NAT has been shown through in vitro and in silico studies to have anti-oxidant and anti-arthritic activity. This led to the selection of these phytoconstituents for subsequent research. The typical challenges and crucial components in the docking method involve aspects like ligand and receptor conformation, flexibility, and cavity detection, among others [41]. These highlight the difficulties and limitations associated with the underlying theories. The plant merits further validation in animal models to confirm its anti-rheumatic activity in vivo, which may result in a modern drug from this plant.

## 4. Materials and Methods

### 4.1. Sample Collection, Authentication, and Extraction

Fresh harsingar (*Nyctanthes arbor-tristis*) leaves were collected from the campus of GLA University, Mathura, India, and authenticated by the Agharkaar Research Institute in Pune, India. (AUTH-22). For the extraction, shadow-dried leaves were grinded into powder. The phytoconstituents were extracted with hydroethanolic solvent using a reflux apparatus, and the extract was concentrated at 45 °C using a rotary vacuum evaporator (Yamato Scientific Co., Tokyo, Japan) For in vitro tests, the final concentrated extracts were kept at −20 °C.

### 4.2. Preliminary Phytochemical Analysis 

The extract was qualitatively analyzed for alkaloids, flavonoids, phenol, steroids, saponins, and tannins using the techniques outlined by Prashant et al. [42].

### 4.3. Quantitative Estimation of Total Phenols and Total Flavonoid

Using the Kokate method [43], qualitative chemical tests were conducted on extract to identify the different phytoconstituents. The total phenol content was evaluated using the Folin–Ciocalteu reagent following Sadasivam and Manikam’s method [44], and the total flavonoid content was estimated using the aluminum chloride method [45]. 

### 4.4. Liquid Chromatography Parameters

LC–MS analysis was performed on Acquity Ultra Performance Liquid Chromatography equipped with a XEVO- TQD interfaced via an ESI source (Waters Co., Milford, CT, USA). The compounds were separated on a Thermo Scientific™ ACCUCORE C-18, 150 × 2.1 mm, 2.6 µm reverse-phase column at a constant flow rate of 0.25 mL/min. An applied column and the auto-sampler temperatures were 35 ± 5 °C and 25 ± 5 °C, respectively. The mobile phase consisted of three solvents. Acetonitrile (A) and 0.1% formic acid buffer was prepared in 95:5 *v*/*v*, and water/ acetonitrile (B) using a multi-step gradient was applied, as shown in Table 6, with a sample injection volume of 2 μL.

#### Mass Spectrometry Parameters

For qualitative analysis, full scan data were acquired in both ES ± ion mode within a mass range of *m*/*z* 150–2000, and the ESI parameters are summarized in Table 7. The LC–MS data acquisition was carried out using Masslynx software version 4.1.

### 4.5. In Vitro Scavenging Potential Assays

The researchers used 2,2-diphenyl-1-picrylhydrazyl (DPPH) free radical scavenging activity and hydrogen peroxide scavenging to test the anti-oxidant power of NAT extracts.

#### 4.5.1. DPPH Free Radical Scavenging Activity

A total of 3 mL of a 0.135 mM DPPH solution was mixed with 20 g/mL, 40 g/mL, 60 g/mL, 80 g/mL, and 100 g/mL of NAT extract. The absorbance (Abs) at 517 nm was measured after 20 min of incubation and compared to the control. As the dose of DPPH went up, its ability to dispose of radicals was shown by the fact that its absorbance at 517 nm went down [46].

(Abs con − Abs sample)/(Abs con) × 100 = %Inhibition Activity (1)

Abs sample is the absorbance of the sample extraction, and Abs con is the absorbance of the control.

#### 4.5.2. Scavenging of Hydrogen Peroxide

Phosphate-buffered saline containing a 20 mM solution of hydrogen peroxide was prepared (PBS, pH 7.4). Hydrogen peroxide solution in PBS (2 mL) was mixed with extract or standard in ethanol (1 mL) at various concentrations. The absorbance was taken after 10 min at 230 nm [47]. Percentage inhibition was calculated for a range of extract concentrations and compared to that of ascorbic acid, the gold standard.

Equation (1): Abs sample is the absorbance of the sample extraction, and Abs con is the absorbance of the control.

### 4.6. In Vitro Anti-Arthritic Activities

The effect of *Nyctanthes arbor-tristis* extract on arthritis was tested, and the results were compared to those of standard anti-inflammatory drugs (diclofenac).

#### 4.6.1. Protein Denaturation Inhibition Assay

The reaction mixture obtained 2.4 mL of 5% bovine serum albumin and 1 mL of an NAT hydroethanolic extract. The pH was changed to 6.3, and the mixture was left to sit at 37 °C for 20 min. After that, the samples were heated at 57 °C for 30 min. After the samples were chilled, a total of 5 mL of phosphate-buffered saline (pH 6.3) was added to each tube. A spectrophotometer set to 660 nm was used to measure turbidity and compare the results to those obtained from the control sample. The percent inhibition of protein denaturation was determined using Equation (1) [48].

#### 4.6.2. Membrane-Stabilization Assay 

To the 4.5 mL assay reaction mixtures, 1 mL of 0.15 M PBS (pH 7.4), 2 mL of hypotonic saline solution (0.25% NaCl), and 1 mL of an aqueous solution containing *Nyctanthes arbor-tristis* were added. The HRBC suspension was 10% *v*/*v* in normal saline. In the control group, 1 mL of hypotonic saline was used because RBCs were not present. The combination was kept in an incubation tank at a temperature of 57 °C for 30 min. The absorbance of the supernatants was measured at 560 nm after they were centrifuged and cooled. The percentage membrane-stabilizing potential was determined using Equation (1) [49].

### 4.7. Splenocyte Proliferation Assay

200 µL of spleen cells at a concentration of 2 × 10^6^ cells/well were used to assess the effect of an extract made from NAT leaves on the growth of splenocytes in a laboratory setting. Using RPMI-1640 medium supplemented with 10% fetal bovine serum (FBS), these cells were cultured in triplicate. Con A concentrations ranging from 1 µg/mL to 20 µg/mL were tested in order to find the best concentration for splenocyte culture. 

The NAT leaf extract was then applied in triplicate to wells of a plate at various concentrations, including 50 g/mL, 100 g/mL, 250 g/mL, 500 g/mL, and 1000 g/mL. The culture plate was then kept in a CO_2_ incubator for 72 h at 37 °C with a 5% CO_2_ atmosphere. A total of 20 L of MTT solution (5 mg/mL) was added to each well after the incubation period. The MTT was reduced, and crystals of formazan were produced. The plate was re-incubated for a total of 4 h at 37 °C with 5% CO_2_ and 80% relative humidity in a CO_2_ incubator. The supernatant was taken out after incubation, and the plate was left to air dry. The formazan crystals were then dissolved in each well by adding 100 L of DMSO. Specifically, an ELISA reader was used to measure the optical density (OD) of the wells at dual wavelengths of 560 nm and 670 nm.

### 4.8. In Vitro Determination of NAT Extract on Cytokine Production

Normal spleen cells were isolated and cultured in the presence of Con A (10 µg/mL) to determine the in vitro effects of the hydroethanolic extract of NAT leaves. Spleen cells were cultured in the wells of their respective rows with NAT leaf extract fractions at various concentrations (50 g/mL, 100 g/mL, 250 g/mL, 500 g/mL, and 1000 g/mL). Supernatants were collected for the quantitative estimation of TNF-α /IL-10 cytokine after a 48 h incubation of spleen cell culture. TNF-α and IL-10 cytokine concentrations in spleen cell culture supernatants were assessed in accordance with the kit instructions offered by B.D. Bioscience (Franklin Lakes, NJ, USA).

### 4.9. In Silico Analysis

For the purpose of visualizing ligand–receptor interactions in Discovery Studio 2021, Python Molecular Version 1.5.7 was used. This program prepared and docked proteins and ligands, generated grids, and produced graphical representations of the interactions between the molecules. The ligand’s 2D structure was downloaded as SDF from PubChem (PubMed (nih)), and the receptor structure was obtained from the RCSB PDB (RCSB PDB: Homepage).

#### 4.9.1. Preparation of Protein

A PDB file containing the three-dimensional structure of cyclooxygenase 2 (COX-2) protein (PDB ID: 1CVU) and tumor necrosis factor (TNF-α) protein (PDB ID: 6RMJ) was obtained from the Protein Data Bank (RCSB PDB: Homepage). The Python Molecular Version was used for the protein preparations (PMV-1.5.7). Bonding arrangements were established after the addition of a heavy hydrogen atom. Even water molecules were eliminated. The structure was finally optimized and reduced in size using a force field [50]. The Procheck server (https://servicesn.mbi.ucla.edu/PROCHECK/, accessed on 1 July 2023) was used to evaluate the quality and conformations. The Ramachandran plot not only interprets the energetically permitted and disallowed conformations of a protein’s structure, but also allows for the visualization of the dihedral angles phi and psi of the amino acid residues present in the protein structure.

#### 4.9.2. Preparation of Ligand and Receptor Grid Generation

The 3D structure generator used the software package PyMOL 2.5 (pymol.org) to display the different conformers of the ligand’s 3D structure. Python Molecular Edition was used to put together the structures (PMV-1.5.7). Grid restricts its active site, where ligands dock, to condensed regions of a receptor protein. By default, Glide creates a grid with a Van der Waals radius of 2.0 and a charge cut-off of 0.50 before applying the force field [51]. For the docking test, a 24 × 24 × 24 cubic box was generated around the active site, and its volume was adjusted so that it had the same volume as the grid box.

Before molecular docking, the 3D SDF structure of ligands was obtained from the PubChem database, and the format conversion was performed via Open Babel GUI-2.4.1 software to transfer the mol2 format required for molecular docking. The protein structures downloaded from the PDB database were stripped of their ligands by PYMOL software. All of the above preparations were conducted, then, in AutoDockTools 1.5.7, AutoDockVina molecular docking simulations were performed, and the binding energies of each protein and ligand were calculated 10 times; of course, the lowest binding energy is the result we need [52]. Next, in this study, the visualization and analysis of the interaction between the two were completed in PYMOL software and the Protein-Ligand Interaction Profiler (PLTP).

#### 4.9.3. Pharmacology Analysis and Preclinical Trials 

Predictions of absorption, metabolism, distribution, excretion, and toxicity were made using pkCSM (predicting small-molecule pharmacokinetic properties using graph-based signatures) for both flavonoids and alkaloids. Analyses of bioactivity, drug similarity, and synthetic accessibility were also a part of the pharmacology analysis in this study. Preclinical studies predicted things like the maximum safe daily dose [53].

#### 4.9.4. PASS (Prediction of Activity Spectra for Substances) Prediction Study 

PASS (Prediction of Activity Spectra for Substances) was only made for the two ligands that inhibited the MAP dephospho-coenzyme A kinase (DPCK) protein at the receptor level the best. We conducted PASS using canonical SMILES from PubChem (http://pubchem.ncbi.nlm.nih.gov/, accessed on 1 July 2023), which is based on the PASSWay 2 drug server (http://www.pharmaexpert.ru/passonline/, accessed on 1 July 2023) [54]. Only when the probability “to be active” (Pa) is greater than 70% can predictions using PASS be considered to be of high quality [55]. A subset of ligands’ biological activity and potential negative effects were predicted by PASS. The ProTox-II server was used to calculate the LD50 and toxicity class (http://tox.charite.de/protox-ii/, accessed on 1 July 2023) [56].

## 5. Conclusions

The active ingredients of NAT is beneficial in the prevention and treatment of arthritis from the in vitro and in silico studies. NAT has anti-inflammatory and anti-oxidant effects. The mechanism of anti-arthritis effects might relate to the regulation of the inflammatory pathway with cyclooxygenase 2 (COX-2) and tumor necrosis factor (TNF-α) altered. The molecular docking analysis and bioactivated study have also showed that Arborside C, Calceolarioside A, carotenoid, nyctanthic acid and oleanoic acid in NAT are the main components that exert anti-inflammatory activity. This work may contribute to the discovery of a new anti-arthritic agents for the use of NAT, which may ease the burden of the joint inflammation patients.

## Figures and Tables

**Table 1:** Chromatographic and mass spectrometry data of identified compounds.

S. No.	RT	Mol. Mass	ES+		ES−				Ref.	
			[M + H]^+^	[M + Na]^+^	[M − H]^−^	[M + Cl]^−^	[M + HCOO]^−^	[M + 2H]^−^		Compound Name
1	6.7	610		633	609	645			Figure 1A,B	Unknown
2	7.4	478			477			955	Figure 1C	Calceolarioside A
3	10.4	510	511	533		545	555		Figure 1D,E	Arborside C
4	12.1	536	537	559			581		Figure 1F,G	Carotenoid
5	13.3	630	631	653			675		Figure 1H,I	Unknown
6	24.1	456			455				Figure 1J	Nyctanthic Acid/ Oleanolic Acid

**Table 2:** Effect of *Nyctanthes arbor-tristis* on splenocyte proliferation. Results are expressed a mean ± SEM.

S. No.	Conc. of Con-A in µg/mL	Conc. Of NAT Extract (µg/mL)	Mean Absorbance ± SEM at 540 nm	Stimulation Index (%)
1.	10	Nil	0.503 ± 0.006	-
2.	10	50	0.689 ± 0.023	36.97
3.	10	100	0.718 ± 0.011	42.74
4.	10	250	0.783 ± 0.018	55.66
5.	10	500	0.528 ± 0.012	4.97
6.	10	1000	0.318 ± 0.017	−36.77

Abbreviations: Concentration concanavalin A (Con A); (µg/mL) microgram per milliliter; ± SEM (standard error of the mean).

**Table 3:** Effect of in vitro exposure of rat splenocytes to different concentrations of *Nyctanthes arbor-tristis* flowers on TNF-α and interleukins-10 cytokines induction.

S. No.	Concentration	Mean Absorbance ± SEM of TNF-α	Mean Absorbance ± SEM of IL-10	Stimulation Index (%) of TNF-α	Stimulation Index (%) of IL-10
1	Control	43.73	1358.12	-	-
2	50	64.76	1434.69	48.09	5.63
3	100	57.81	1593.91	32.19	17.36
4	250	53.95	2158.79	23.37	58.95
5	500	49.81	2330.74	13.9	71.61
6	1000	47.42	2235.84	8.43	64.62

Abbreviations: standard error of the mean (±SEM); tumor necrosis factor alpha (TNF-α); interleukin 10 (IL-10).

**Table 4:** ADME properties of phytoconstituents.

Properties	Arborside C	Calceolarioside A	Carotenoid	Nyctanthic Acid	Oleanoic Acid
Pgb-substrate	Yes	Yes	Yes	No	No
GI absorption (Gastrointestinal Absorption)	Low	Low	Low	Low	Low
BBB (Blood -Brain Barrier)	No	No	No	No	No
CYP450 1A2 inhibition	No	No	No	No	No
CYP450 3A4 inhibition	No	No	No	No	No
CYP450 2C9 inhibition	No	No	No	Yes	No
CYP450 2C19 inhibition	No	No	No	No	No
CYP450 2D6 inhibition	No	No	No	No	No
Skin permeation	−9.51 cm/s	−8.80 cm/s	−1.14 cm/s	−2.45 cm/s	−3.77 cm/s
Bioavailability Score	0.11	0.17	0.17	0.85	0.85
Synthetic accessibility	6.14	5.20	5.82	5.73	6.08

Abbreviations: P-glycoprotein (Pgb-substrate); cytochrome P450 family 1 subfamily A polypeptide 2 (CYP450 1A2); cytochrome P450 family 3 subfamily A polypeptide 4 (CYP450 3A4); cytochrome P450 family 2 subfamily C polypeptide 9 (CYP450 2C9); cytochrome P450 family 2 subfamily C polypeptide 19 (CYP450 2C19); cytochrome P450 family 2 subfamily D member 6 (CYP450 2D6).

**Table 5:** Prediction of LD50, predicted toxicity class, and activity spectra for substances (PASS) of phytoconstituent for anti-rheumatic activity.

Phytoconstituents	Predicted LD50	Predicted Toxicity Class	Pa	Pi	Activity
Arborside C	2000 mg/kg	4	0.798	0.007	Anti-inflammatory
			0.738	0.012	Immuno-suppressant
Calceolarioside A	5000 mg/kg	5	0.946	0.001	Free radical scavenger
			0.716	0.04	Anti-oxidant
Carotenoid	4000 mg/kg	5	0.746	0.011	Immunosuppressant
Nyctanthic acid	11,800 mg/kg	2	0.772	0.009	Anti-inflammatory
Oleanoic acid	2000 mg/kg	4	0.819	0.005	Anti-inflammatory
			0.814	0.002	Nitric oxide antagonist

Abbreviations: LD50 (lethal dose 50%); Pa (probability “to be active”); Pi (probability “to be inactive”).

**Table 6:** A multi-step linear gradient qualitative analysis.

S. No.	Time	Flow Rate (mL/min)	Solvent A (Acetonitrile)	Solvent B (0.1% Formic Acid in 95:5 *v*/*v* Water/Acetonitrile)
1.	0.1	0.25	5	95
2.	1.0	0.25	5	95
3.	10.0	0.25	30	70
4.	14.0	0.25	60	40
5.	16.0	0.25	60	40
6.	24.0	0.25	80	20
7.	32.0	0.25	80	20
8.	35.0	0.25	5	95
9.	40.0	0.25	5	95

Abbreviations: milliliters per minute (mL/min); volume per volume (*v*/*v*).

**Table 7:** ESI parameters.

Source Temperature	120 °C
Desolvation Temperature	350 °C
Capillary	3.5(kV)
Cone	30V
Cone Gas Flow	50 (L/h)
Desolvation Gas Flow	950 (L/h)

Abbreviations: electrospray ionization (ESI); (L/Hr) Liter per hour; (kV) kilovolt *(*°C).

## Data Availability

The data supporting the findings of this study are available from the corresponding author upon reasonable request.

## Sample Availability

Samples of the compounds are available from the authors.
